# Supplementary material for: A subset of SMN complex members have a specific role in tissue regeneration via ERBB pathway-mediated proliferation
Source: NPJ Regen Med. 2020 Mar 25;5:6. doi: 10.1038/s41536-020-0089-0 (PMC7096462; doi:10.1038/s41536-020-0089-0)
Supplement: Supplementary file 1 — supplemental figures and tables [file 41536_2020_89_MOESM1_ESM.pdf]

Suppl. Figure 1

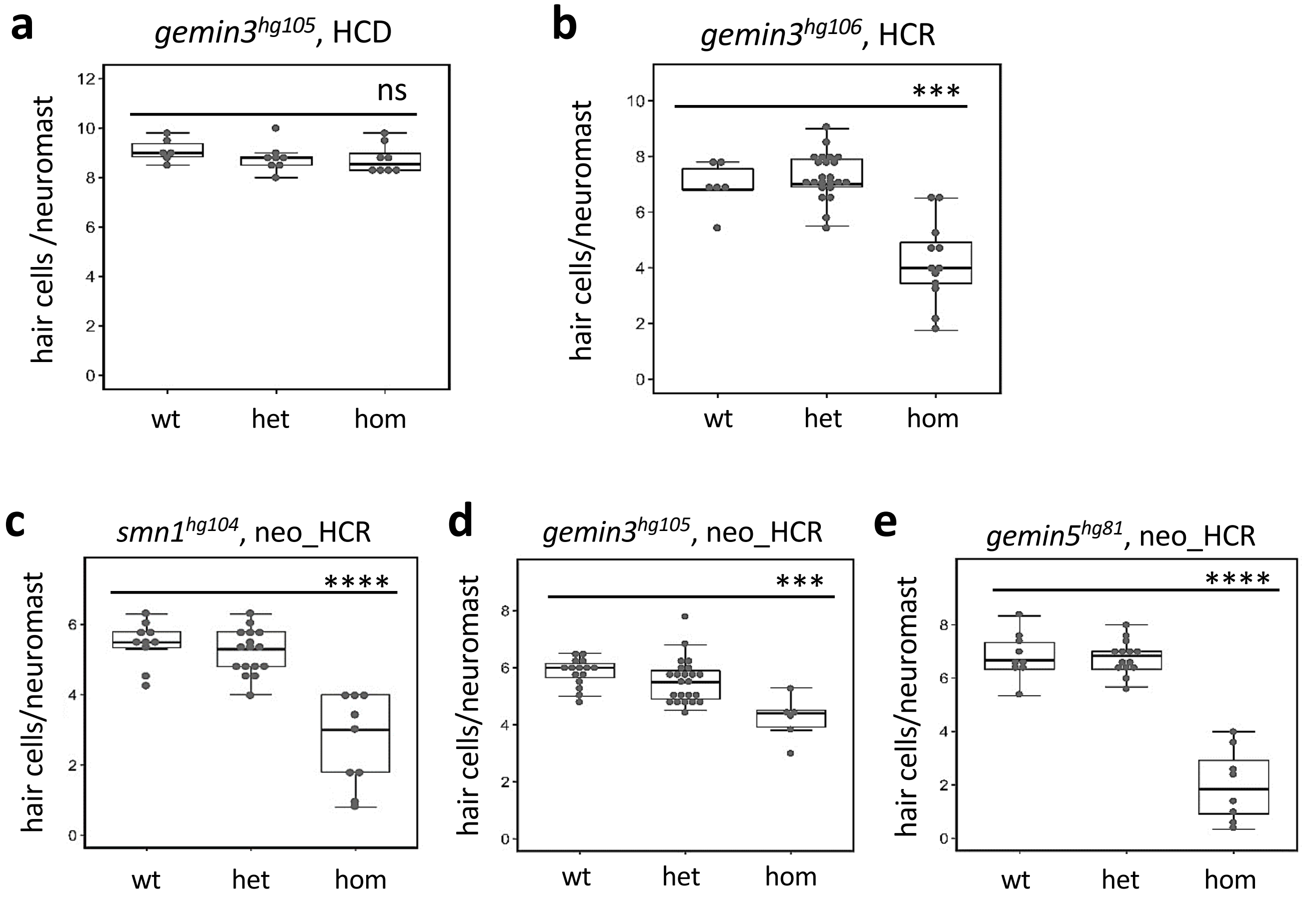

Suppl. Fig. 1 Hair cell development and regeneration in *gemin3*, *gemin5*, and *smn1* mutants. (A) Normal hair cell development in *gemin3*^{hg105} mutants. (B) Impaired hair cell regeneration in *gemin3*^{hg106} mutants. (C-E) impaired hair cell regeneration after neomycin-induced hair cell ablation in homozygous mutations of *smn1*^{hg104} (C), *gemin3*^{hg105} (D) and *gemin5*^{hg81} (E). HCD, hair cell development. HCR, hair cell regeneration. neo, neomycin. wt, wild-type. het, heterozygotes. hom, homozygotes. Error bars in the graphs represent mean ± s.e.m. ns, $P > 0.05$; ***$P < 0.001$; ****$P < 0.0001$. Approximately 40 embryos were used for each of the analyses and then genotyped to determine the genotype-phenotype correlation.

Suppl. Figure 2

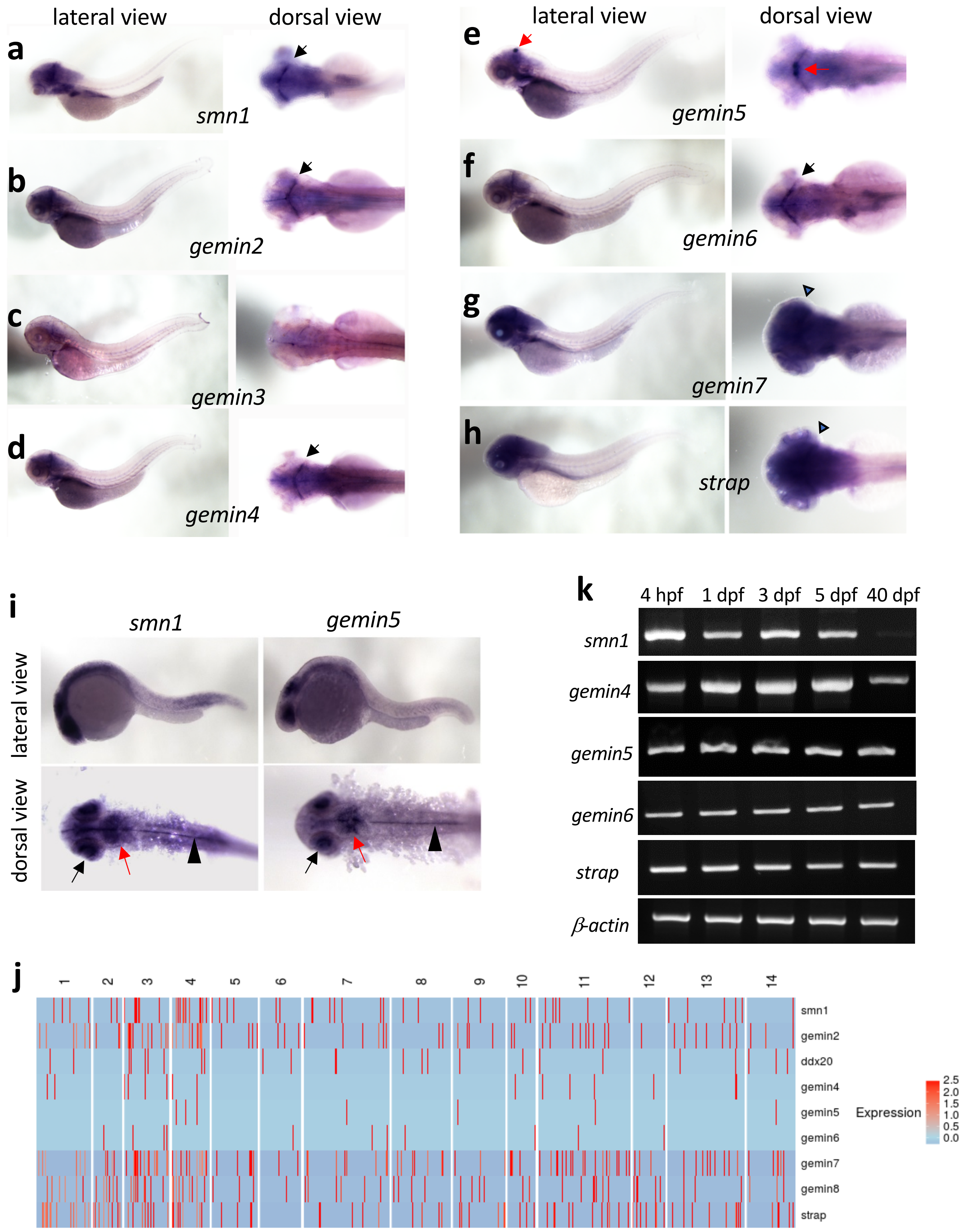

Suppl. Fig. 2 Expression analysis of the SMN complex genes.

(A-H) Whole-mount in situ hybridization analysis using TAB5 wild-type embryos at 3 dpf to detect the mRNA expression patterns of *smn1* (A), *gemin2* (B), *gemin3* (C), *gemin4* (D), *gemin5* (E), *gemin6* (F), *gemin7* (G) and *strap* (H). Black arrows point to the restricted brain expression of *smn1*, *gemin2*, *gemin4*, and *gemin6*. Red arrows point to the unique expression of *gemin5* as a stripe with a thickening in the midline. Black arrowheads point to the ubiquitous expression of *gemin7* and *strap*. No obvious expression of *gemin8* was detected at this embryonic stage. (I) Whole-mount in situ hybridization analysis using TAB5-wild type embryos at 1 dpf to detect the mRNA expression patterns of *smn1* and *gemin5*. For imaging the dorsal view, yolks of the embryos were removed for better visualization of the staining. Black arrows point to expression in the eye. Red arrows point to the expression in the forebrain. Black arrowheads point to midline expression. For whole-mount in situ hybridization analysis, each probe was used for 15 wild-type embryos. (J) Heatmap of mRNA expression of the SMN complex genes in the scRNA-sequencing data reported by Lush ME, et al [17]. Each red hash indicates a cell that expresses the specific gene. The numbers 1-14 indicate the different clusters of neuromast cells, with clusters 1 and 2 indicating hair cells, clusters 5 and 6 indicating mantle cells, and the others indicating support cells. The *gemin5* gene is not expressed in hair cells (clusters 1 and 2). The expression of *ddx20*/*gemin3*, *gemin4*, *gemin5* and *gemin6* are overall weaker than the other genes. (K) Semi-quantitative PCR analysis of the mRNA expression levels of the SMN complex genes at different developmental stages. All gels were derived from the same experiment and were processed in parallel.

Suppl. Figure 3

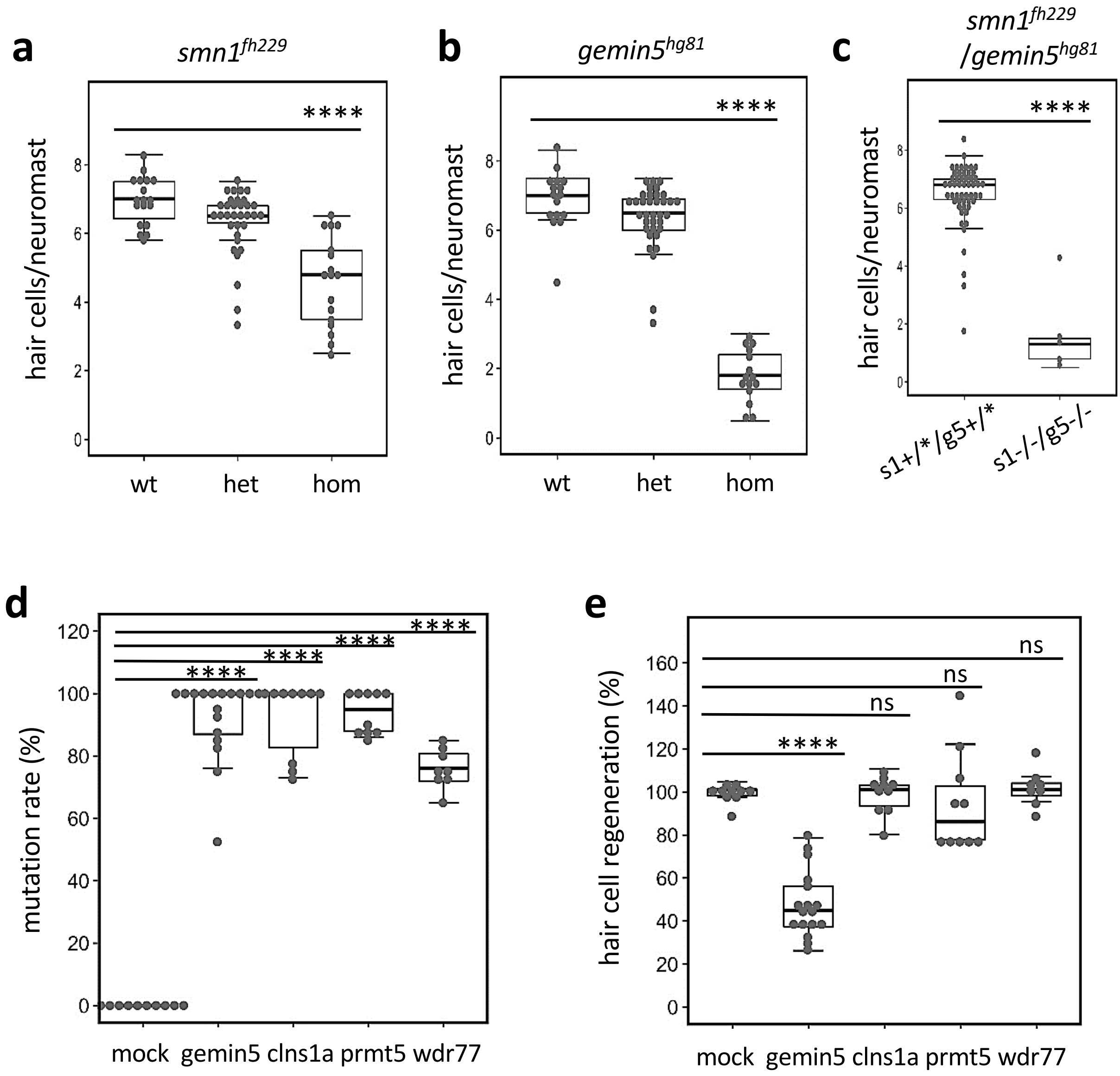

Suppl. Fig. 3 Hair cell regeneration in *smn1^{fh229}/gemin5^{hg81}* double mutants and the PRMT5 complex mutants.

(A-C) Analysis of the *smn1^{fh229}/gemin5^{hg81}* double mutants. The 130 embryos used for the analysis were generated from a single pair of parents, each carrying heterozygous mutations for both *smn1^{fh229}* and *gemin5^{hg81}*. Genotyping results showed 5/130 embryos were double mutants. Graphs show the hair cells regenerated from the *smn1^{fh229}* mutant (A), *gemin5^{hg81}* mutant (B), and *smn1^{fh229}/gemin5^{hg81}* double mutant (C). The difference is significant between the *smn1* wild-type and homozygotes, between the *gemin5* wild-type and homozygotes, and between the *smn1/gemin5* control and double mutant (**** P < 0.0001 for all three groups). There is no difference between the *gemin5* homozygotes and the *smn1/gemin5* double homozygotes (ns, P > 0.05. Not labeled in the graphs). (D-E) Analysis of the PRMT5 complex mutants. Mutation frequency shown in (D) was measured by the CRISPR-STAT method. Hair cell regeneration in (E) was normalized to Cas9 injected mock embryos. Approximately 10 embryos were used for generating each data point.

Suppl. Figure 4

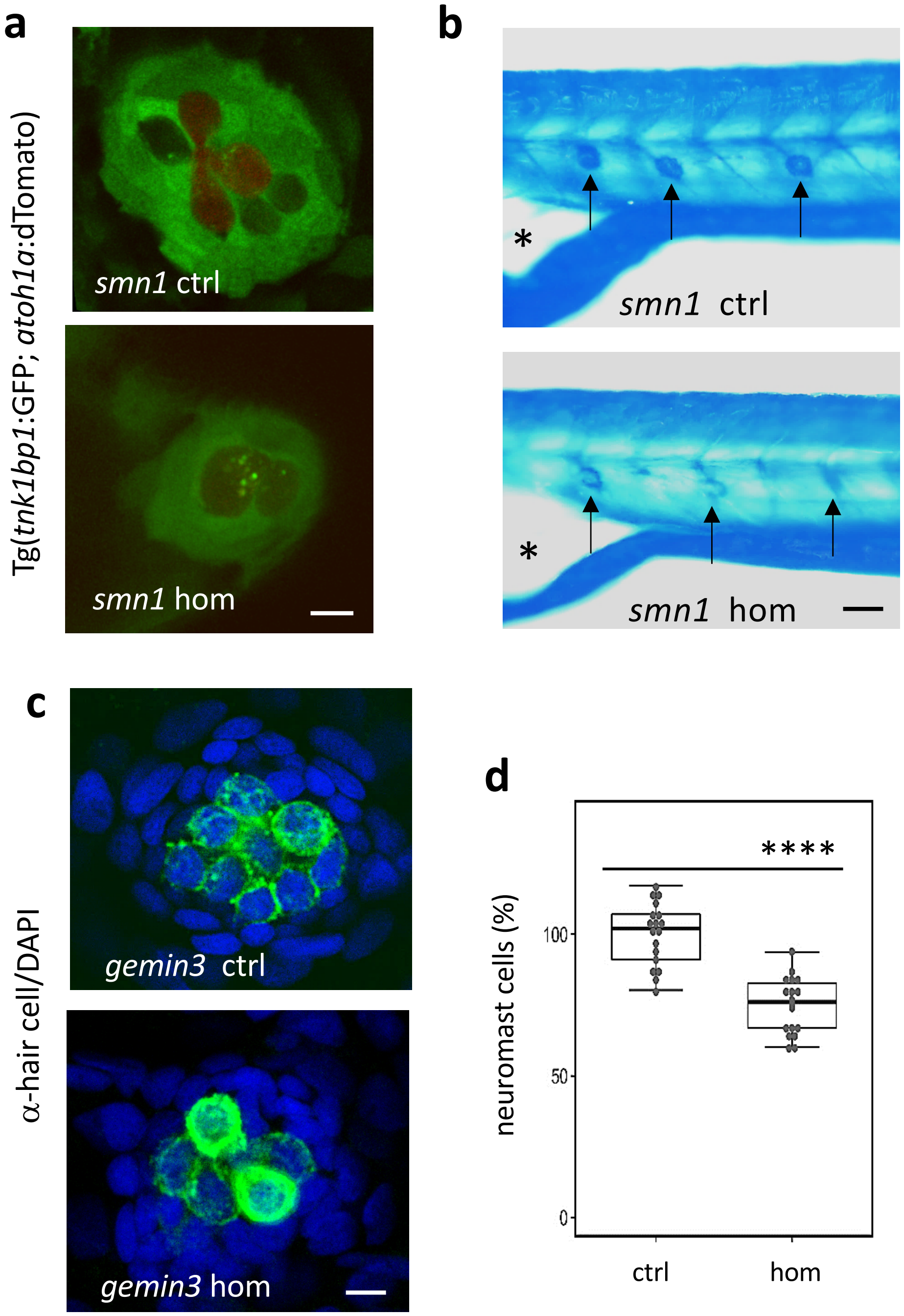

Suppl. Fig. 4 Smaller neuromasts observed in *smn1*^{fh229} and *gemin3*^{hg105} mutants at 2 days post hair cell ablation.

(A) Imaging the lateral line neuromasts in the *smn1*^{fh229} control and mutant embryos at 2 days post hair cell ablation, using Tg(*tnks1bp1*:GFP) to label the support cells and Tg(*atoh1a*:dTomato) to label the hair cells. Scale bar, 10 μm. (B) Alkaline phosphatase staining of lateral line neuromasts in the *smn1*^{fh229} control and mutant embryos at 2 days post hair cell ablation. Arrows point to the neuromasts. Asterisks label the yolk sac. Scale bar, 50 μm. (C) Confocal images of lateral line neuromasts in the *gemin3*^{hg105} control and mutant embryos at 2 days post hair cell ablation. Neuromasts were stained with anti-hair cell antibodies (green) and DAPI (blue). Scale bar, 10 μm. (D) Quantification of neuromast cells in the *gemin3*^{hg105} control and mutant embryos. Error bars in the graphs represent mean ± s.e.m. A significant reduction is found in the neuromast cells (**** $P < 0.0001$). The numbers are presented as percentages based on cell numbers in confocal images. Each data point was generated from 18 embryos.

Suppl. Figure 5

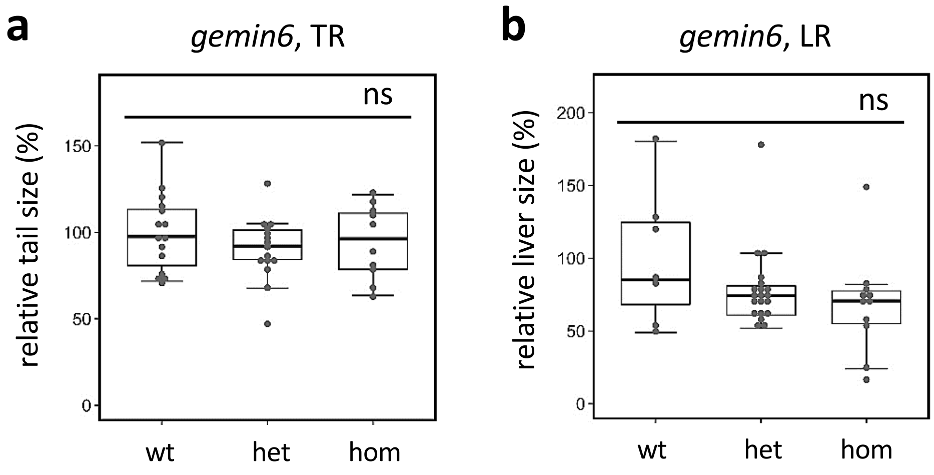

Suppl. Fig. 5 The *gemin6*^{hg110} mutation has no impact on the regeneration of caudal fins or livers. (A) Quantification of the area of the regenerated tail fins. (B) Quantification of the area of the regenerated livers. Liver is labeled by Tg(*fabp10a*:CFP-NTR). TR, tail regeneration. LR, liver regeneration. Graphs show the mean ± s.e.m. The difference between the wild-type (wt) and homozygous mutants (hom) were not significant (ns, $P > 0.05$). The results were obtained from analyzing approximately 40 embryos generated from a heterozygous incross (for fin regeneration), or heterozygous incross in the background of the transgenic Tg(*fabp10a*:CFP-NTR) (for liver regeneration).

Suppl. Figure 6

a

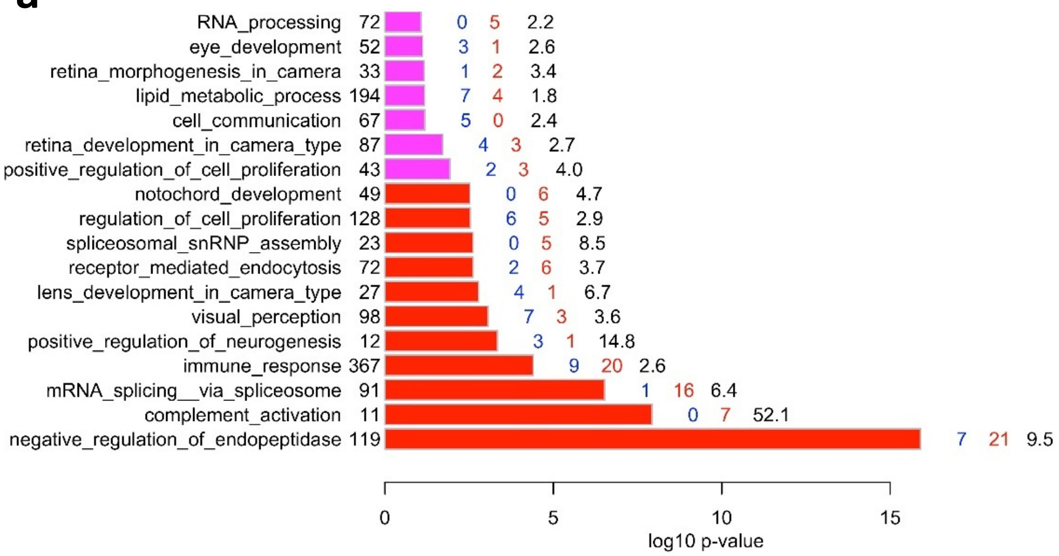

b

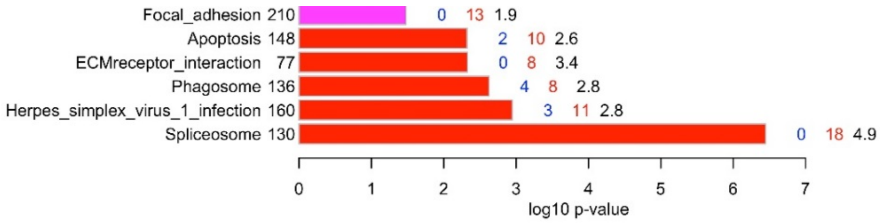

Suppl. Figure 6

c

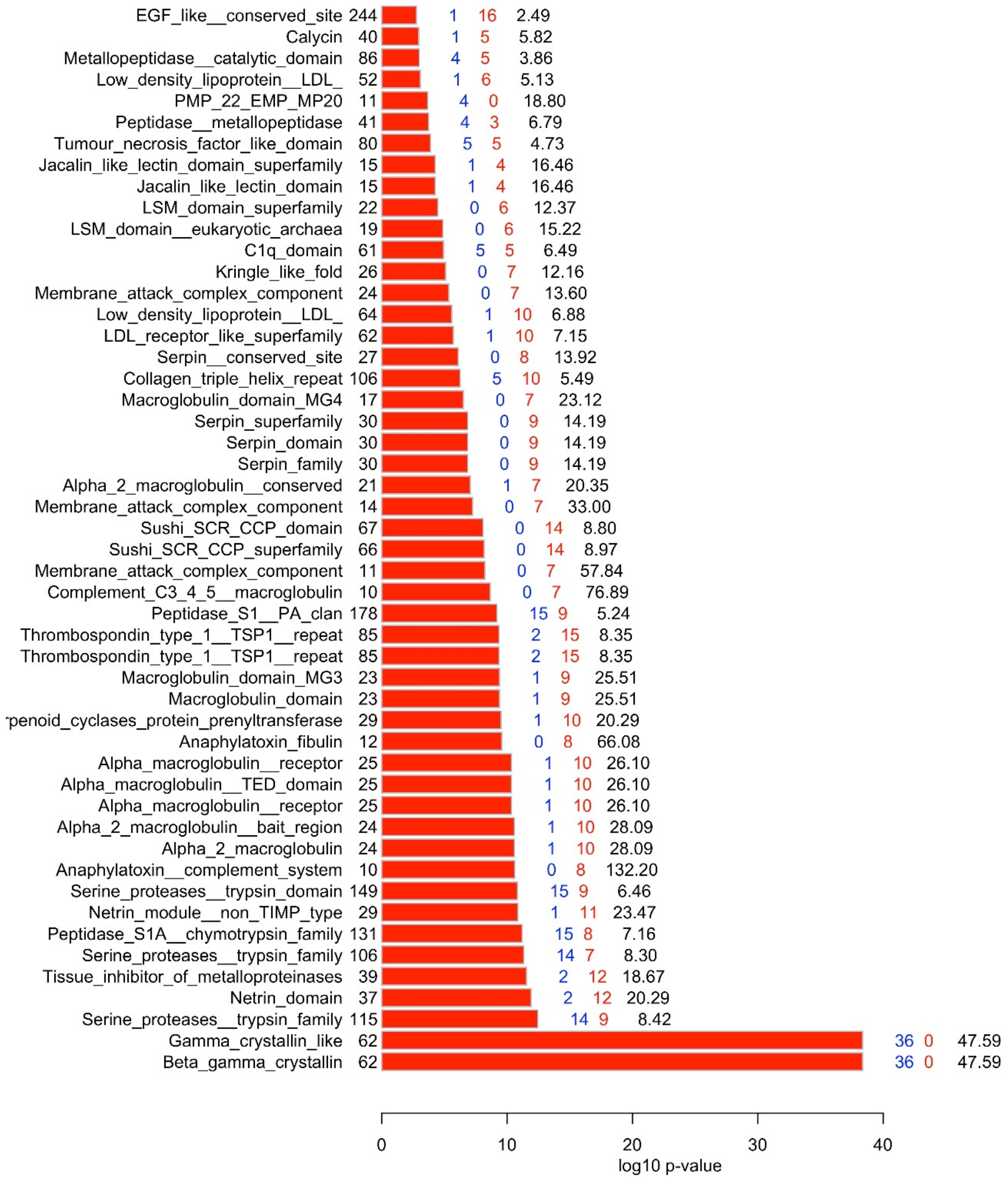

Suppl. Fig. 6 RNA-Seq data analysis reveals regeneration-associated molecular programming. (A) Regeneration-associated gene ontology biological processes. (B) Regeneration-associated KEGG pathways. (C) Regeneration-associated IPR protein families, domains and functions. The analysis was done by comparing the regeneration gene mutants to the non-regeneration gene mutants. The numbers after the molecular programs shows the size of the gene set. Numbers after the bars: blue, the number of down-regulated genes in the regeneration gene mutants; red, the number of up-regulated genes in the regeneration gene mutants; black, the odds ratio of the Fisher's exact test. Bar colors: red, p-value<0.01; magenta, p-value<0.1.

Suppl. Figure 7

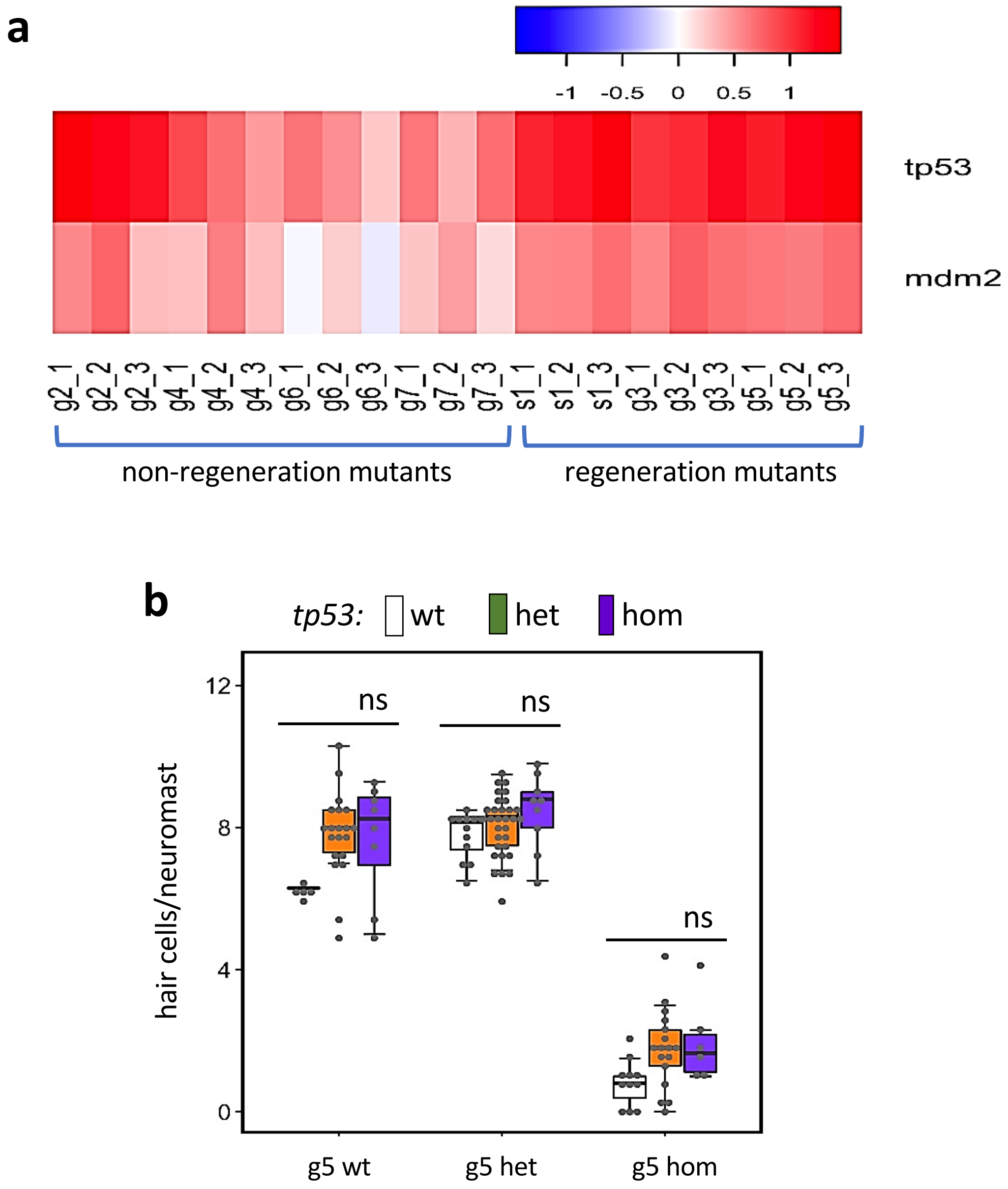

Suppl. Fig. 7 *tp53* knockdown has no impact on regeneration in the *gemin5* mutant.
(A) Heat map of *tp53* and *mdm2* mRNA expression in the RNA-sequencing samples. (B) Hair cell regeneration analysis for the *tp53*^{zdf1}/*gemin5*^{hg81} double mutants. Graph shows the mean ± s.e.m. The *tp53* ^{zdf1} mutation caused no difference in the wild-type (wt), heterozygotes (het), or homozygotes (hom) of *gemin5* embryos (ns, P > 0.05). The data are generated from analyzing 119 embryos generated from a pairwise incross of double heterozygous parents. After hair cell regeneration analysis, all 119 embryos were genotyped and 6 of them carried homozygous mutations for both *tp53*^{zdf1} and *gemin5*^{hg81}.

Suppl. Figure 8

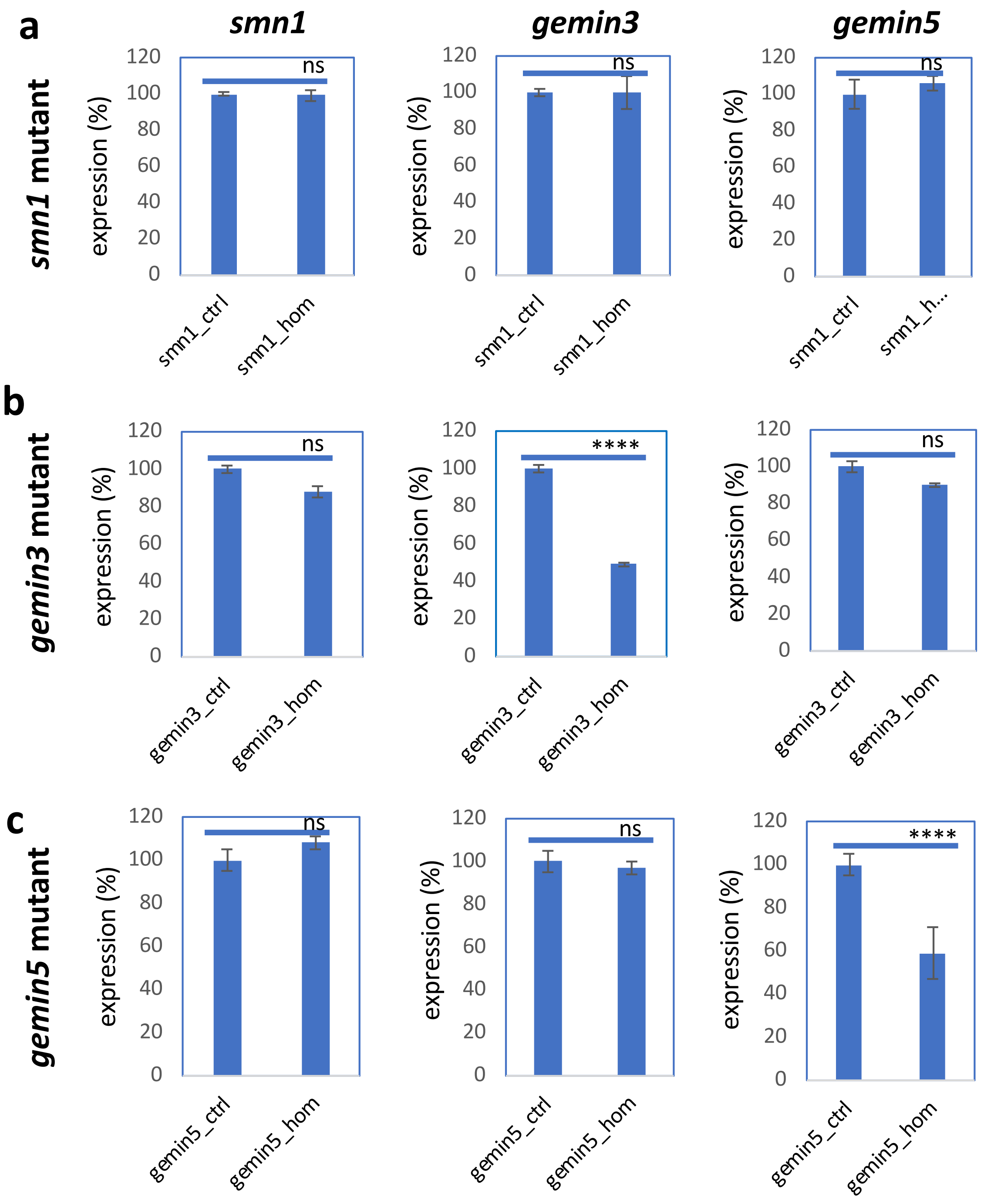

Suppl. Fig. 8 Expression of *smn1*, *gemin3* and *gemin5* in the control and mutant embryos of *smn1*^{fh229} (A), *gemin3*^{hg105} (B) and *gemin5*^{hg80} (C).
The graphs show the expression of the mRNA of the SMN complex genes, with the data obtained from RNA-Seq analysis. Genes examined are labeled on the top. Mutants used are labeled on the left. Graphs show the mean ± s.e.m. ns, $P > 0.05$; ****$P < 0.0001$.

Suppl. Figure 9

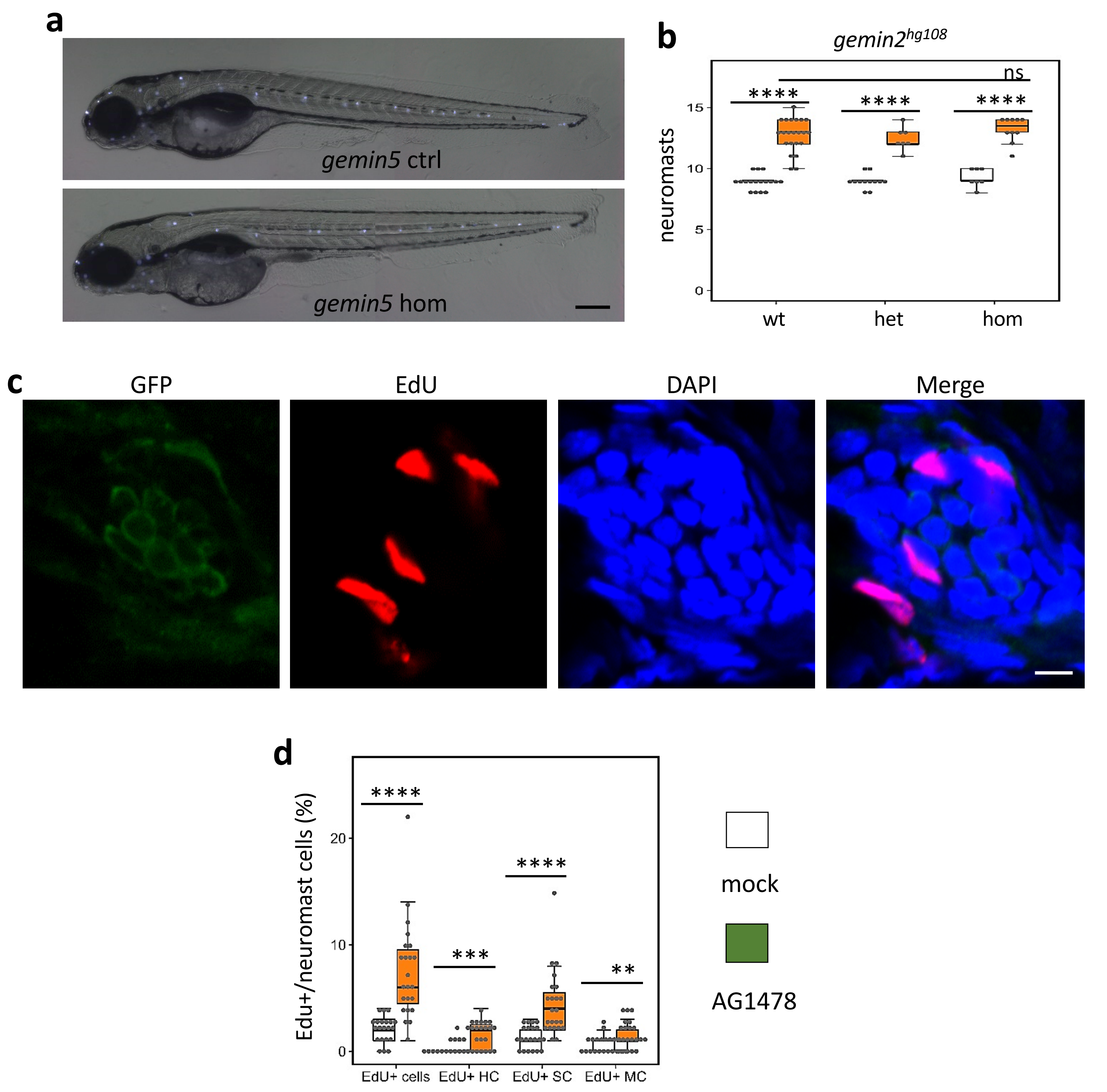

Suppl. Fig. 9 ErbB pathway inhibitor AG1478 promotes lateral line neuromast cell proliferation.

(A) Neuromasts in the untreated control and *gemin5*^{hg81} mutant embryos at 5 dpf. Neuromasts are shown as white dots. No clear difference observed in between. Scale bar, 250 µm. (B) Neuromasts in the untreated and AG1478-treated *gemin2*^{hg108} mutant. Approximately 40 embryos generated from a heterozygous incross were used for each condition. (C) Confocal images of a lateral line neuromast of a TAB5 embryo at 5 dpf labeled by transgenic GFP from Tg(*pou4f3*:GAP-GFP) and Tg(*SqET20*:EGFP), EdU, DAPI and the merged. Representative images are shown. Scale bar, 10 µm. For the EdU experiment, the embryos were treated with 2 µM AG1478 from 1 – 4 dpf and then used for EdU labeling. Scale bar, 10 µm. (D) Quantification of the proliferating cells in neuromasts (EdU+_all), hair cells (EdU+_HC), support cells (EdU+_SC), and mantle cells (EdU+_MC) in the mock and AG1478-treated TAB5 embryos at 5 dpf. Each data point was generated from analyzing 22 embryos. Error bars represent mean ± s.e.m. Statistical difference is labeled as ***P < 0.001 and ****P < 0.0001.

Suppl. Figure 10

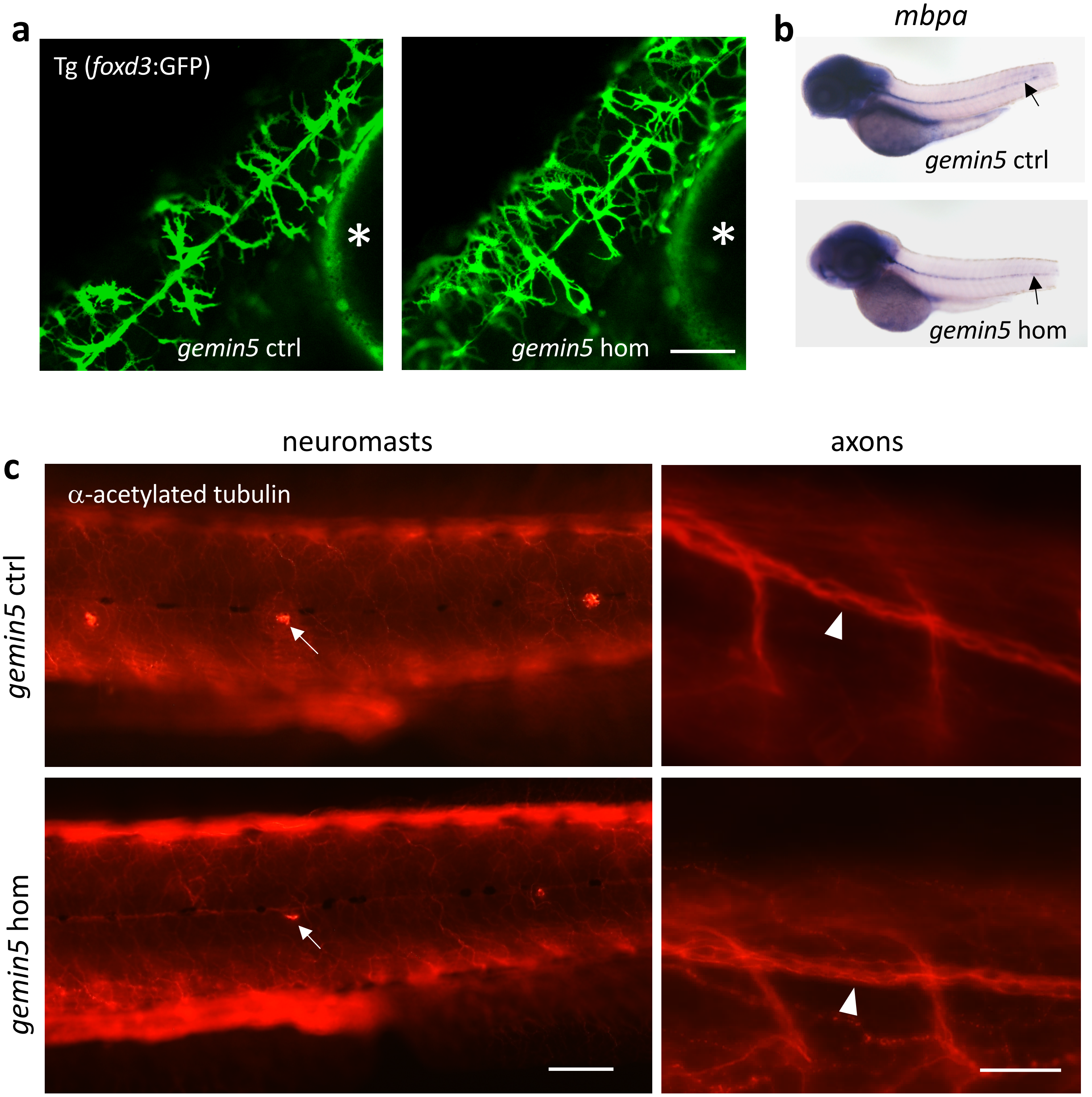

Suppl. Fig. 10 Normal development of Schwann cells and lateral line axons in the *gemin5*^{hg81} mutant.

(A) Confocal images of Schwann cells in the lateral line of the control and *gemin5*^{hg81} mutant at 2 dpf. Schwann cells are labeled by Tg(*foxd3*:GFP) transgene. Asterisks label the embryonic yolk. Scale bar, 100 µm. (B) Whole-mount in situ hybridization analysis of *myelin basic protein a* (*mbpa*) mRNA expression in the control and *gemin5*^{hg81} mutant at 4 dpf. Arrows point to the expression in the lateral line. The caudal fin folds were amputated to obtain tissue for mutation genotyping. (C) Fluorescent images of the lateral line axons labeled by anti-acetylated-tubulin antibodies in the control and *gemin5*^{hg81} at 2 days post hair cell ablation. Representative images are shown. Arrows point to lateral line neuromasts. Arrowheads point to the lateral line axons. Scale bar, 100 µm.

Suppl. Table 1 Genetic mutations used in this study.

Genes	Mutations	References
smn1	Y262X	fh229, [70]
smn1	2 bp insertion	hg104, this study
gemin3	10 bp deletion	hg105, this study
gemin3	9 bp deletion	hg106, this study
gemin5	2 bp deletion	hg107, this study
gemin5	1 bp deletion	hg80, [14]
gemin5	20 bp deletion	hg81, [14]
gemin2	7 bp deletion	hg108, this study
gemin4	11 bp insertion	hg109, this study
gemin6	14 bp deletion	hg110, this study
gemin7	1 bp deletion	hg111, this study
gemin8	7 bp insertion	hg112, this study
strap/unrip	8 bp deletion	hg113, this study
nrg1	19 bp insertion	hg114, this study
erbb3b	7 bp deletion	hg115, this study
tp53	M214K	zdf1, [33]
tp53	7 bp deletion	hg91, [34]

Suppl. Table 2 Mutations in seven of the nine SMN complex members affect adult survival.

Genes	Mutations	Fish # _total	Fish#_wt	Fish#_het	Fish#_hom
smn1	Y262X	20	7	13	0
gemin2	7 del	19	7	12	0
gemin3	9 del	20	8	12	0
gemin4	11 ins	27	10	17	0
gemin5	1 del	25	8	17	0
gemin6	14 del	34	9	25	0
gemin7	1 del	26	9	17	0

Suppl. Table 3 Treatments used for screening the biological pathways that contribute to the regeneration phenotype.

Treatments	Pathways/Activities	Doses	References
Cold	cell proliferation	4°C, at 4 dpf for 8 hours	[58]
Heat	cell proliferation	34°C, from 1.5 - 5 dpf	[58]
Antimycin A	energy production	0.1 ng/ml, from 3 - 5 dpf	[71,72]
Cycloheximide	translation	1 µg/ml, from 1 - 5 dpf	[73]
AG1478	ErbB	2 µM, from 1 - 5 dpf<br>For HCR, 2.5 or 5 µM from 5-7 dpf	[35]
DAPT	Notch	10 µM, from 1 -5 dpf<br>For HCR, 50 µM from 5 -7 dpf	[74]
Dexamethasone	immunity	For HCR, 10 µM from 5 -7 dpf	[75]
Prednisolone	immunity	For HCR, 10 µM from 5 - 7 dpf	[75]
1-azakenpaullone	Wnt	1 µM, from 1 -5 dpf<br>For HCR, 3 µM from 5 - 7 dpf	[76]
BIO	Wnt	0.3 µM, from 1 - 5 dpf	[35]
IWR-1	Wnt	60 µM, from 1 - 5 dpf	[35]
SU5402	FGF	5 µM, from 1 - 5 dpf	[35]
SB505124	TGFb	0.1 µM, from 1 - 5 dpf	[77]
H2O2	oxidative stress	0.01%, from 1 - 5 dpf	[78]
NAC	antioxidants	100 µM, from 1 - 5 dpf<br>For HCR, 200 µM from 5-7 dpf	[79,80]
GSH	antioxidants	100 µM, from 1 - 5 dpf<br>For HCR, 200 µM from 5-7 dpf	[79,80]

Supplemental Data set 1

Significant transcriptional changes in each sibling-mutant pair identified by RNA-seq.
S1= smn1, G2=gemin2, G3= gemin3, G4=gemin4 G5=gemin5, G6=gemin6, G7=gemin7

Supplemental Data set 2

Misspliced genes in each sibling-mutant pair identified by RNA-seq. Genes highlighted in red are the genes showing a difference in both expression level and splicing.

S1=smn1, G2=gemin2, G3=gemin3, G4=gemin4, G5=gemin5, G6=gemin6, G7=gemin7, all=merge of all 7 datasets
